# Factors influencing the accuracy for tissue classification in multi spectral in-vivo endoscopy for the upper gastro-internal tract

**DOI:** 10.1038/s41598-020-60389-5

**Published:** 2020-02-26

**Authors:** Martin Hohmann, Heinz Albrecht, Benjamin Lengenfelder, Florian Klämpfl, Michael Schmidt

**Affiliations:** 1 Friedrich-Alexander-Universität Erlangen-Nürnberg (FAU), Institute of Photonic Technologies (LPT), Konrad-Zuse-Straße 3/5, 91052 Erlangen, Germany; 2 Erlangen Graduate School in Advanced Optical Technologies (SAOT), Paul-Gordan-Straße 6, 91052 Erlangen, Germany; 3 Department of Internal Medicine II, Kliniken des Landkreises Neumarkt i.d.OPf., Nürnberger Str. 12, 92318 Neumarkt, Germany

**Keywords:** Imaging and sensing, Biophotonics

## Abstract

Hyper spectral imaging is a possible way for disease detection. However, for carcinoma detection most of the results are *ex-vivo*. However, *in-vivo* results of endoscopic studies still show fairly low accuracies in contrast to the good results of many *ex-vivo* studies. To overcome this problem and to provide a reasonable explanation, Monte-Carlo simulations of photon trajectories are proposed as a tool to generate multi spectral images including inter patient variations to simulate 40 patients. Furthermore, these simulations have the huge advantage that the position of the carcinoma is known. Due to this, the effect of mislabelled data can be studied. As shown in this study, a percentage of 30–35% of mislabelled data might lead to significant decrease of the accuracy from around 90% to around 70–75%. Therefore, the main focus of hyper spectral imaging has to be the exact characterization of the training data in the future.

## Introduction

Gastric cancer is the second most frequent cause of cancer related deaths worldwide^1^. Despite a wide development of new technologies in the recent years, high definition white light endoscopy (HD-WLE) is still considered as state of the art by official endoscopy guidelines. One of the possible alternatives is virtual chromoendoscopy^2^ for which meta analyses^3^ prefer this method above all others.

Moreover in their review, Swager *et al*.^4^ concluded that spectroscopic quantitative measurements of tissue need further investigation. It is expected that they may improve the methodology of virtual chromoendoscopy further. The high accuracy of virtual chromoendoscopy provides a hint that extracted fine spectral features allow a better accuracy than normal HD-WLE.

One way of spectroscopic measurement is multi/hyper spectral imaging (MSI/HSI). So far, both have been proven successful in finding cancer in different parts of the human body such as: larynx^5^ with a sensitivity of 55–87% and specificity of nearly 100%^6^, cell analysis from cervix^7^ with a sensitivity ranging from 66 to 100%, breast^8,9^, colon^10–17^ or oesophagus/stomach^18–22^. Thereby, the group from Sakaida reached an accuracy of 79% in the first study^19^ and 85% in their second study^20^ in an *ex-vivo* setting. However, the transfer of these results to *in-vivo* by our previous publications showed significant worse results. An accuracy of 64% is reached in the first study^21^ and an accuracy of 68% in the second study^22^. These improvements could be reached by using elaborated methods for data analysis. In summary, the *in-vivo* results^22^ are outperformed by around 20 per cent points by the good *ex-vivo* classification results^20^.

To overcome this issue, this study tries to find the reason which might explain the limited quality of *in-vivo* multi/hyper spectral classification results with the focus on endoscopic problems. For this, multi spectral images are simulated by the means of Monte-Carlo-Simulations (MCS). The huge advantage of using simulations is the fact that in comparison to *in-vivo* experiments the precise margin of the carcinoma is known. Therefore, it can be tested what effect the wrong margin has on the final classification results. In comparison in *in-vivo* cases the margin of the carcinoma is not precisely known^23^. Therefore, a certain part of the data will be labelled wrongly. Moreover, the *in-vivo* data set does not provide a controlled environment.

## Materials and methods

### Parameter for the Monte Carlo simulation

To generate multi spectral images, an own graphical-processing-unit (GPU)-based MCS-software is used. The software was validated successfully against the diffusion approximation and Monte-Carlo-extreme (MCX)^24^. One graph for validation is shown in the attachment in figure A. In general, the tissue inhomogeneities are disregarded by the MCS.

For the simulation, a carcinoma in oesophageal tissue is simulated due to the fact that the optical properties (*μ*_*a*_, *μ*_*s*_ or g) of these tissue types is known. The optical properties are taken from the study from Holmer *et al*.^25^ instead of the own optical properties^26^. The data from Holmer *et al*.^25^ is used due to the fact that Holmer *et al*.^25^ measured the optical properties from carcinoma and from healthy oesophagus. However, there is a high chance that the measurements from Holmer *et al*.^25^ have a systematic error as shown by Hohmann *et al*.^26^. Despite the fact that they use cryo-homogenisation, this should not influence the optical properties^27^. Thus, the source of the potential error cannot be explained. Nevertheless, it can be expected that healthy tissue and the carcinoma tissue have a similar systematic error. Therefore, it makes more sense to use the dataset from Holmer *et al*.^25^ since at least the difference between both tissue types might be realistic.

To derive the optical properties and include inter patient variations, the following strategy is chosen: First, a centre value is calculated and, second, the variations are derived. The centre value of the optical properties are derived by averaging the optical properties for each spectral bandwidth, using the spectra of the illumination source as weight from our older clinical study for carcinomas in the stomach^21^. This is done for each wavelength band to ensure that values match our the previous publication^21^. By integrating the optical properties abbreviated with OP times the normalized intensity (I) of the light source, the final optical properties are calculated as weighted mean:

1 $$O{P}_{average}=\frac{{\int }_{{\lambda }_{a}}^{{\lambda }_{b}}I(\widehat{\lambda })\cdot OP(\widehat{\lambda })d\widehat{\lambda }}{{\lambda }_{b}-{\lambda }_{a}}$$

Thereby, *λ* is the wavelength and *λ*_*b*_ and *λ*_*a*_ are the boundaries at which the intensity is zero. In total, the following seven centre wavelengths are simulated: 396, 437, 474, 511, 549, 574 and 637 nm.

The variation of the optical properties is taken from the standard deviations from Holmer *et al*.^25^ as it is the only data available. Due to the fact that Holmer *et al*.^25^ show only three values for the standard deviation, the missing data points of the standard deviation are interpolated. First, the relative standard deviation is calculated. A linear fit is used to generate the data for the wavelengths used in this study. This step is done as the data is required. An optimal solution would be to switch to Bayesian statistics to generate a-posteriori information as we showed it for diffuse reflectance spectroscopy^28^.

For the simulation, it is assumed that most of the standard deviation of the optical properties is caused by the inter patient variations from the study from Holmer *et al*.^25^ and not from random variations of the measuring procedure. Due to the fact that they dissociated the oesophagus, the intra patient variations should be much lower than they are normally.

The inter patient variations are derived by using a relative standard deviation from the mean values and varying it with a Gaussian distribution with the same full width at half maximium (FWHM) as the relative standard deviation. It should be noted that this assumption might not be true because most likely the distribution of the optical properties is not Gaussian distributed. However due to the fact that Holmer *et al*.^25^ assumed the Gaussian distribution, the same assumption has to be done in this study. Furthermore, it is expected that the variation of *μ*_*a*_, *μ*_*s*_ and g might not be independent from each other. However to date, there is no knowledge of the dependent behaviour and therefore this effect has to be neglected.

For the simulation, the variations of the optical properties cannot be seen as wavelength independent. For example, a variation of the scattering for blue light is not independent of a variation of the scattering for red light. Normally, a change of scattering in one wavelength range implies a change of the scattering in another wavelength range. However, as the functional dependence between different wavelengths is not known, an additional random fluctuation should be considered to allow wavelength dependencies. For the scattering, this might be caused by different sizes of the scatterers.

As the functional dependencies are not known, a more practical approach is chosen. The effect of the same relative variations across all wavelengths and a completely random fluctuation is considered. The same relative variation of the scattering coefficient and/or the g-factor would lead to a different penetration depths and the tissue would appear brighter or darker without major changes of the tissue colour. The same relative variation of the absorption coefficient across all wavelengths would have a similar effect compared to the scattering. However, the largest difference would be for the wavelengths with a high absorption coefficient. As in the tissue, blood as red absorber is present, the tissue would appear more or less reddish and, therefore, the amount of blood in the tissue would be varied with this. Both variations are realistic and definitely play a major role in the inter patient variations.

In contrast, if the optical properties are varied randomly for each wavelength, the colour of the tissue would change independent which from the three optical properties is varied. This effect happens only slightly in healthy tissue as for most healthy tissue the colours are similar. But they are not the same. However for cancerous tissue, this effect might play a bigger role due to the fact that in the upper gastro-intestinal tract, carcinomas might appear reddish to discoloured^19^.

To simulate both effects, for healthy tissue 80% of the variation is assumed to occur due to the same relative variations across all wavelengths. This effect should not alter the colour significantly nevertheless it should allow small fluctuations between the patients. For the cancerous tissue, only half of the variations is assumed to occur due to the same relative variations across all wavelengths. This allows the the appearance of discoloured carcinomas. However, it should be noted that there is no way available currently to derive these numbers with a higher precision than with educated guessing. Nevertheless, this effect should be considered as carcinomas might appear reddish to discoloured^19^ and healthy tissue appears more similar between patients. Box plots of the final optical properties are shown in the attachment B, C and D.

**Figure 1:**
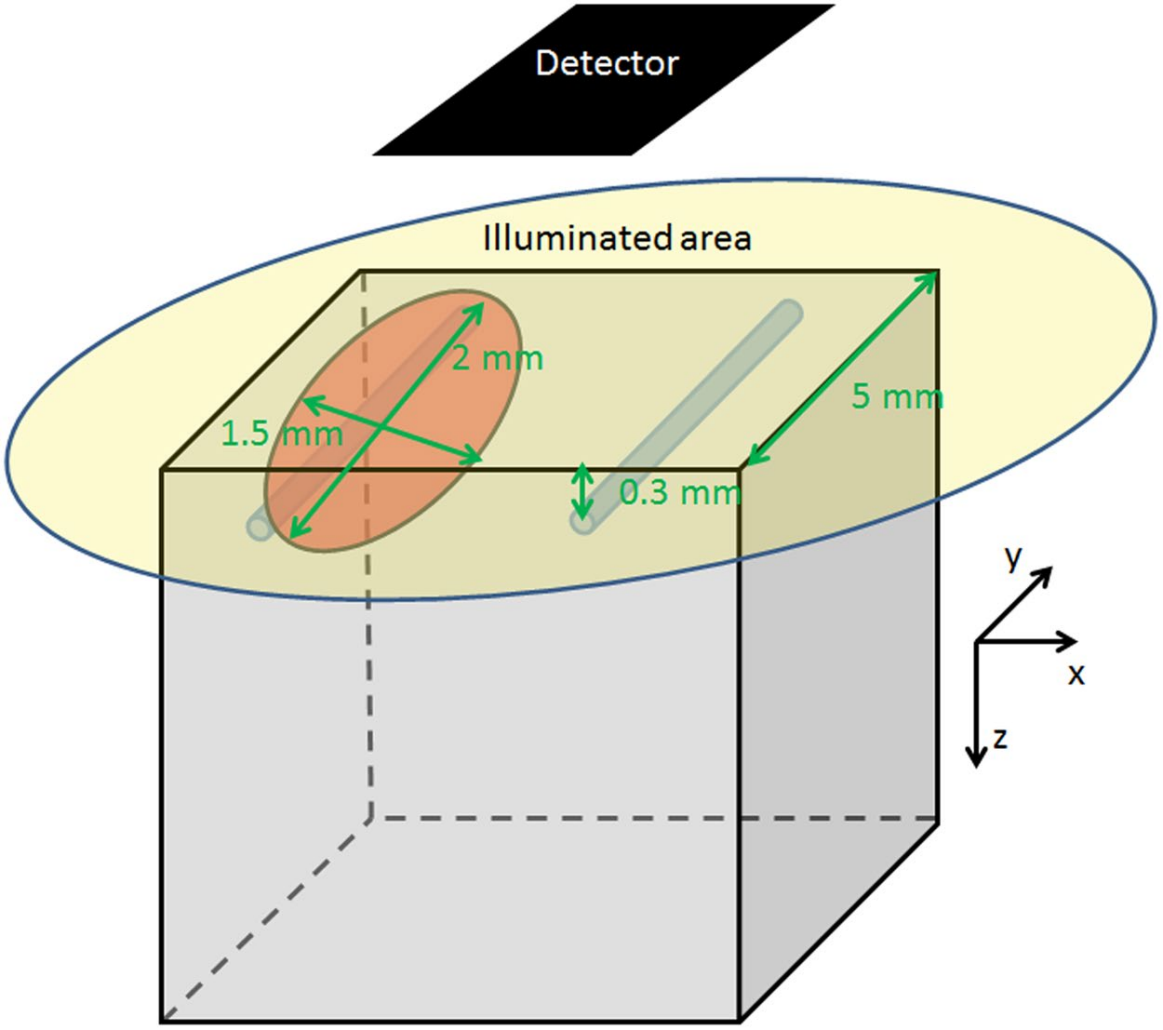
Set-up of the Monte-Carlo simulations. Due to the fact that the upper surface is divided into nine equally sized squares, this sketch shows the inner square. Position of the vessels and the carcinomas are symbolic to the real ones for better visibility. Also their size increased for the same reason.

After the optical properties are set, the geometric set-up is the next thing to consider. The set-up for the MCS is illustrated in Fig. 1. The upper surface is divided into nine equally sized squares. This is done as only photons from the inner square are considered for detection. Furthermore, only in the centre square the carcinoma is placed. Thus, this sketch in Fig. 1 shows the inner square. The whole set-up has a volume of 15 × 15 × 15 *mm*^3^ as bulk tissue. The simulation volume is chosen to be small for two reasons: First to speed up the simulation process. Second, there is an overlap area at boundary at the carcinoma where its effect on the back reflection fades out more the further away you are from boundary. For for bigger carcinomas, the optical overlap area between healthy and cancerous area would be relatively smaller. Therefore the effect of mislabelling would be much stronger. Thus, smaller carcinomas are chosen. Moreover, for small carcinomas mislabelling is more likely. Only a single layer structure is simulated due to the fact that Holmer *et al*.^25^ dissociated the oesophagus before performing their measurement.

The simulation volume includes two blood vessels as cylinders and the cancerous tissue as ellipsoid. Two blood vessels are added to have a little bit of inhomogeneities in the tissue. The two blood vessels have a diameter of 10 microns. One vessel is 0.25 mm below the centre of the carcinoma and the second one is placed 0.3 mm below the surface. The carcinoma has a diameter of 1.5, 2.0 and 0.1 mm in x, y and z-direction. The position of the centre is 6.5, 7.5 and 0.4 mm from the left upper edge. The tissue is simulated with semi-infinite boundary conditions. Hence, Fresnel reflection is only taken into account on the top surface. The simulation stops when 131’072’000 photons reach the detector. Less than 5% of the photons reach the detector and therefore at least 3 billion photons have to be simulated.

For the simulation, close imaging is simulated. Even if in *in-vivo* situation the imaging is often not perpendicular, this set-up is chosen due to three reasons: First, it is easier to simulate. Second, if a tilted simulation is chosen, there are too many possible ways to choose from. This would make it difficult to compare if different people show different angles. Third, the imaging is done with a small distance between endoscope and surface. In this case, it is also in *in-vivo* situations possible to have nearly perpendicular imaging. The light source emulates an endoscopic light source with homogeneous intensity distribution on the surface. The light in the centre hits the surface perpendicular and the light in the outer part with an angle of 30 degrees. Hence, the light source is a point source with a distance of about 1.7 mm to the surface. For the detection the surface is divided into nine squares with equal size. The photons are only taken from the central square to minimize boundary effects from the finite simulation volume. There are no other photons which stray in the detector due to the fact that only the photons from this central square are checked if they can reach the detector. All others are immediately terminated. The surface of the central square is divided into 301 times 301 pixels which is imaged on the detector.

For the 131’072’000 photon, 1446 photons arrive in average per pixel. Hence, the resulting noise is expected to be high. The noise can be estimated by the Binomial distribution of the photons. Equation 2 shows the formula to calculate the standard deviation if it is assumed that all pixels have the same probability that a photon arrives:

2 $$std=\sqrt{n\cdot p\cdot (1-p)},$$

where n is the total amount of photons (*n* = 131’072’000) and p is the probability for hitting a single detector which is *p* = 1.1037 ⋅ 10^−5^. Thus, the resulting standard deviation is 38 intensity values for each detector, resulting in an average noise of about 3% of the signal. Therefore, a further noise reduction step is required. For the noise reduction, a seven times seven pixel wide Gaussian filter with a FWHM of 2 pixel is used. The noise can be estimated in the same way as before but with 49 times less pixels. This results in an average signal of *signal* = (70890 ± 270) photons, reducing the noise to around 0.4%. However due the usage of the Gaussian filter, the noise is expected to be higher. Stronger noise reduction or more photons are not needed due to the fact that the noise level is similar or even better than in our previous *in-vivo* study^21^. Using the Gaussian filtering instead of decreasing the spatial resolution of the detector has two advantages: First, the resolution is similar to at least older endoscopes. Second, the later introduced shifts of the carcinoma can be done with the necessary precision. Reducing the resolution by the same factor would lead to a pixel size of 43 times 43 pixels. This is too less to adequately do the later calculations. With the finalized set-up of the Monte-Carlo simulation 40 patients are simulated and for each patient one endoscopic image is generated. Each patient has a randomly selected different set of optical properties for the carcinoma and the healthy tissue.

### Parameter difference between real carcinoma and carcinoma used for training

**Figure 2:**
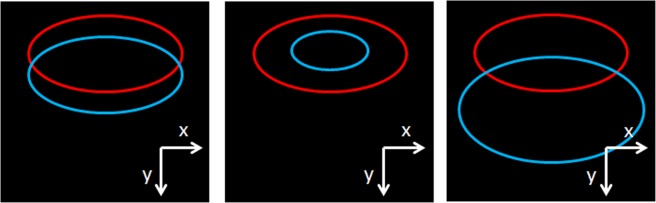
Different models of how to vary the carcinoma data which is used for training. The images represent the final generate image (topview). The red ellipse shows the margin of the carcinoma and the blue ellipse shows the margin of the carcinoma which is used for the training of the classifier. Left: Variation of the position, Middle: Variation of the size, Right: Random variation of size and position.

In this study, the effect of the mismatch between the real carcinoma and the carcinoma used for training is calculated for three different kinds of mismatch. Figure 2 shows an example of the three kinds of mismatch that are investigated in this study. The red ellipse shows the margin of the carcinoma and the blue ellipse shows the margin of the carcinoma which is used for the training of the classifier. Furthermore Table 1 shows an overview of the variations between the carcinoma used for training and the real one.

**Table 1:** Used variations between the real carcinoma and the carcinoma used for training. For the last line, both parameters are varied randomly at the same time.

**Varied Parameter (Unit)**	**Minimum**	**Maximum**	**Step size**
Position (one minus area overlap in%)	0%	50%	5%
Size (size of the real carcinoma in%)	25%	175%	25%
Position and size randomly (size)	25%	175%	
Position and size randomly (position in radius carcinoma)	0	1.25	

First, the effect of the shift between the real carcinoma and the one used for training is tested as symbolized in Fig. 2 left. The carcinomas are shifted in y-direction in a way that the overlapping area between the real carcinoma and the one used for the training of the classifiers is varied from 100 to 50% in steps of five per cent points. This simulates the situation when that part of the carcinoma is found while part of the healthy tissue is seen as carcinoma.

Second, the effect of the relative size of the carcinoma used for training and the real carcinoma is tested as symbolized in Fig. 2 middle. The size of the carcinoma used for training is varied from 0.25 times the area of the real carcinoma to 1.75 times the size of the real carcinoma in steps of 0.25. This simulates that too much of healthy tissue is seen as cancerous or if not the whole cancerous area can be detected.

Third, the random combination of both previous effects is studied as symbolized in Fig. 2 right. In this part, a maximal variation is set. This maximal difference of the area is 0.25 or 1.75 times the area of the real carcinoma. The maximal shift of the carcinoma is 1.25 times the radius of the original carcinoma. For selecting a value, a uniform random number is chosen between the minimum and the maximum difference from the real carcinoma. Before the random number determines the position the maximal shift and size variations are limited from 0 to 100% of the maximal variation in steps of 10 per cent points. Hence, the strength of the random overlap can be varied. For the random combination every patient has a different position of the carcinoma used for training.

The last studied effect is expected to be the most realistic, as sometimes only random parts of the carcinoma can be labelled correctly or way too much of the tissue might be labelled as cancerous. Moreover, for this study it is the most realistic setting due to the fact that not all patients have the same mislabelling. The labelling differs from patient to patient. Sometimes the margin of the carcinoma is pretty good, sometimes only a part of the carcinoma is found and sometimes a big chuck of healthy tissue is seen as cancerous.

However for the third mismatch effect, the real overlap is unknown compared to the other two effects. To estimate the overlap, an average overlap area is calculated by choosing 1000 times a random combination of size and position. However, two different kinds of overlap have to be considered. First, the amount of the original carcinoma which is covered by the one used for training and second the amount of the carcinoma used for training which contains the actual carcinoma. Both parameter describe the same area. Nevertheless, they describe it from a different perspective. The parameters differ when the size of the carcinoma used for training differs. The effect can be seen in Fig. 2 middle and right. For example in Fig. 2 middle the amount of the original carcinoma which is covered by the one used for training is around 30% while the amount of the carcinoma used for training which contains the actual carcinoma is 100%.

**Figure 3:**
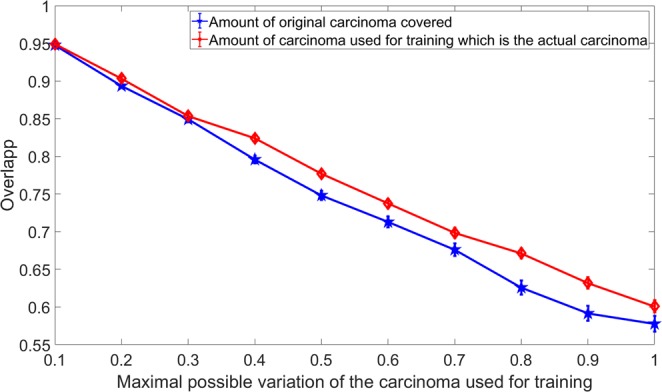
Overlap and the standard error between the real carcinoma and the one used for training. The red graph shows the amount of carcinoma used for training which is actually real carcinoma and the blue graph shows the amount of real carcinoma covered by the one used for training.

 Figure 3 shows both overlaps for different percentage of the maximal variation. It can be seen that both variations differ and therefore only the relative maximal variation will be used as variable. On average, the amount of real carcinoma that is covered is higher than the amount of carcinoma used for training which is actually the real carcinoma. Both graphs drop from 95% overlap to 60–65% overlap. This means even in the worst case, in average around 60–65% of the carcinomas are correctly used for the training. This number seems to be realistic or even underestimates the error as even in the case of correct markings there might be a more than 60% mislabelled area (Fig. 3 from Yoshinaga *et al*.^23^).

### Statistical data evaluation

The statistical data analysis is done the same way as in our previous *in-vivo* study^21^. The data analysis is done pixel based. For training first, the principal component analysis (PCA) is used for feature reduction and the amount of used features is selected in a way that 99% of the variance is used for the analysis. For the training only, one per cent of the data is used to speed up the training process. The data is selected randomly. For classification, RobustBoost (RB), support vector machine (SVM) with a linear and a Gaussian kernel and random forest walk (RFW) are used. The evaluation of the classifiers is done by the leave-one-out method. Therefore, n-1 patients are used for training the left out one is used for testing (n=40). This is done for all possible combinations.

In general, terms such as accuracy are used to measure how close the data is to the training labels. Additionally to this standard way, it is used to compare the classification results to the real carcinoma. This subdivision is necessary as the effect of mislabelling is studied. Therefore, the results have to be compared to the real data set and the one used for training which includes mislabelled data points. For the evaluation of the results the accuracy2 (ACC2) and the Matthews correlation coefficient (MCC)^29^ are used. This way of calculating the ACC2 is robust against the case that the dataset is not symmetric. However, it can be interpreted similar to the standard accuracy. The MCC is chosen due to the fact that it is the best available single value to characterize the classification in a single number^30^. The ACC2 is calculated as follows:

3 $$ACC2=\frac{0.5\cdot TP}{TP+FN}+\frac{0.5\cdot TN}{TN+FP}$$

where TP is the amount of true positives, TN is the amount of true negatives, FP is the amount of false positives and FN is the amount of false negatives. The MCC is calculated as follows^29^:

4 $$MCC=\frac{TP\cdot TN-FP\cdot FN}{\sqrt{(TP+FP)(TP+FN)(TN+FP)(TN+FN)}}.$$

It should be noted that the MCC scales between  −1 and 1 and a value of zero means the classification does not work. This result corresponds to ACC2=50%. Both measures, the ACC2 as well as the MCC are calculated for the position of the original carcinoma as well as the carcinoma used for training.

## Results and discussion

**Figure 4:**
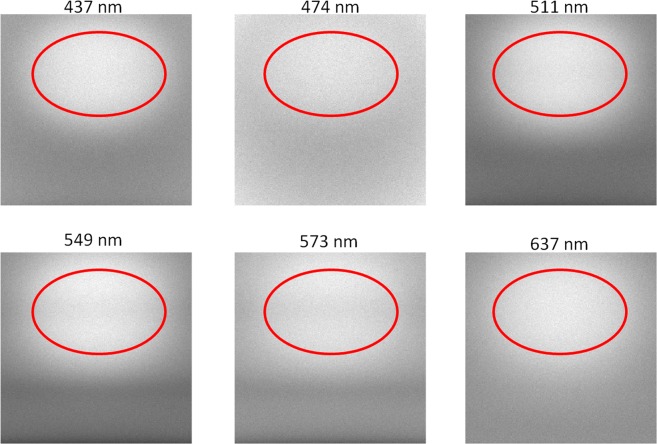
Simulated example image for one patient for six wavelengths. The red ellipses show the position of the carcinoma.

 Figure 4 shows an example for a simulated multi spectral image. The bright part shows the carcinoma. It can be seen that the contrast varies for different wavelengths. Additionally, the blood vessels are only visible for 511–573 nm due to the fact, that for these wavelengths the penetration depth of the light is big enough and furthermore the absorption of the haemoglobin is high enough to generate a contrast.

**Figure 5:**
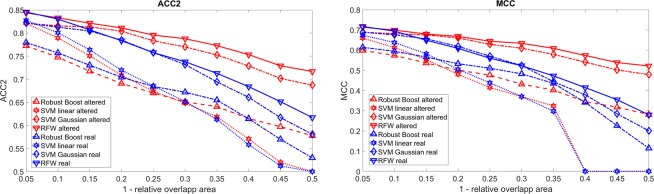
ACC2 and MCC as function of the overlap between the real carcinoma and the one used for training where zero means 100% overlap and 0.5 represents 50% overlap. The red lines represent the detection of the shifted carcinoma and the blue lines that of the real carcinoma.

 Figure 5 shows the effect of the shift of the carcinoma used for training in comparison to the real one. The results for zero shift are similar to the one with a low shift. For all cases, the ACC2 as well as the MCC drop for higher shifts. The ACC2 drops from around 85% to 60–70%, depending on the classifier. The MCC drops from 0.7 to 0.2–0.5 depending on the classifier. For both measures, the real carcinomas are detected worse than the ones used for training. Thus, it might happen in real scenarios that the classifier might be trained on the wrong data set. However, this effect is especially strong in this study due to the usage of small carcinomas for the simulation. For bigger carcinomas, the intermediate area between cancerous and healthy tissue in which the image is effected by both tissue types is relatively smaller. However for early stage carcinomas, it might still play a role in realistic cases.

Moreover, on RFW and SVM with a Gaussian kernel there is the lowest effect due to the mislabelling. However in the previous *in-vivo* study^21^, SVM with linear kernel and RB showed the best results. These results here only describe a constant shift which is not realistic. Despite this, RB seems to consistently find the real carcinoma better than the one used for training for high shifts. Thus, RB should definitely be considered further. Similar results were shown in our previous *in-vivo* study^21^ for carcinomas in the stomach. In all cases, the real carcinoma is found best if the size used for training is chosen correctly or a little bit bigger. This effect happens due to the fact that the back reflectance outside the carcinoma is still partly effected by the carcinoma due to the scattering of the tissue due to the overlap area. As a conclusion for realistic scenarios, it seems to be beneficial to select a little bit higher margin of the carcinoma. Despite the fact that a too big margin might further improve the results of the ACC2 and MCC for the carcinoma used for training, the real carcinoma is found worse. Thus, the carcinoma margin should not be selected too big despite the improvement of the accuracy. For this case, all classifiers show a similar behaviour. RFW again shows the best results.

**Figure 6:**
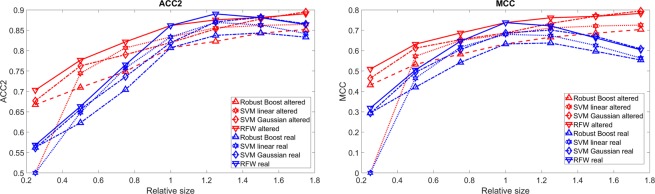
ACC2 and MCC as function of the relative size of carcinoma used for training in comparison to the real one. The red lines represent the detection of the shifted carcinoma and the blue lines that of the real carcinoma. PCA is used for feature selection.

**Figure 7:**
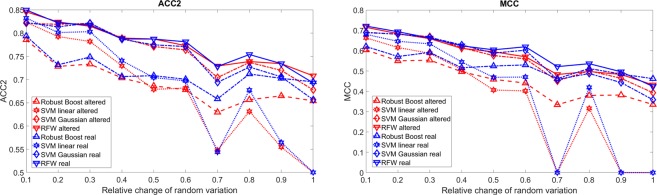
ACC2 and MCC as function of the average overlap shown as percentage of the maximal shift and size variation. The red lines represent the detection of the shifted carcinoma and the blue lines of the real carcinoma. PCA is used for feature selection.

Figure 6 shows the effect of choosing a different size of the carcinoma used for training in comparison to the real one. Figure 7 shows the effect of the random variation of the size and position of the carcinoma. The results for zero shift are similar to the one with a low shift. According to Fig. 3 the maximal relative change is an average overlap of around 60%. Both measures show a decrease of accuracy for stronger mislabelling. The ACC2 drops from 80–85% to 65–70% and the MCC drops from 0.6–0.7 to 0.3–0.4. For around 70% average overlap, the results drop significantly. Without taking mucus or inflammation into account it is possible that the maximal accuracy which can be reached currently is around 70%. Thus for further endoscopic *in-vivo* imaging, the margin has to be found more precise to allow better results. Up to now, there are two solutions: First, Goto *et al*.^20^ used *ex-vivo* oesophagus and did regular biopsies across the whole oesophagus. With this good classification results could be reached. Second, Liu *et al*.^31^ used the complete endoscopic image and the classifier just decided if there is a carcinoma somewhere present in the image. However, they could not show where it is. Again RFW and SVM with Gaussian kernel show the best results. As for the examples before, RB shows better results for the real carcinoma than for the one used for training for the cases of high mismatch.

## Conclusion

In this study, it could be shown that there might be a maximal accuracy for automated classification of *in-vivo* endoscopic multi and potentially hyper spectral images. Despite the fact that the analysis is done for multi-spectral images, the same mislabelling happens for hyper spectral images and thus the results might be transferable. Finer spectral features found by hyper spectral imaging might simplify the discrimination. However, the main absorbers in the UV/VIS/NIR-range such as haemoglobin, fat, water, melanin, proteins have a wide spectral range without narrow spectral features. Hence, hyper spectral imaging is likely to see the same effect. The expected maximal accuracy for *in-vivo* endoscopy is around 70% for a pixel per pixel analysis. To our view, the improvement of this require better knowledge of the margin of the carcinoma which is difficult for *in-vivo* studies. Thus, a very precise optical biopsy technique might enable better classification results.

As a further result some practical conclusions can also be done for *in-vivo* endoscopic classification problems in future: First, while a large marging around the carcinoma increases the classification results of the carcinomas which are used for labelling, the real carcinomas might be found worse. Thus, the margin for classification should only be increased a little bit. As an unexpected result, it can be even seen that RB has the highest MCC for the maximal variation. The MCC for the real carcinoma even outperforms all other classifiers. If this study would not be a simulative study, RB might be rejected for its low MCC. However, the knowledge of the position of the real carcinoma allows to show that is actually finds the real carcinoma. Therefore, in future RB should be included in *in-vivo* endoscopic classification problems and it should be look if the false positives from it are actually carcinomas.

Despite this conclusions, it should be said that this studies has currently a few drawbacks. First, the optical properties, which are used for the MCS, might might have an systematic error. Second for the deviation of the optical properties, a lot things have to be assumed as the required information is not available: e.g. the distribution of the optical properties is assumed to be Gaussian distributed. However from the values from Holmer *et al*.^25^, it can be seen that they are not Gaussian distributed due to the fact the presented standard deviation is nearly as large as the value itself. At the same time, negative absorption and scattering coefficients are not possible for standard tissue. The effect of the assumptions is difficult to assess as the overall variation can be over or underestimated. For assessing the influence, a posteriori information of the optical properties are needed. Without this, even educating guessing is difficult. For the MCS, the comparable simple geometric set-up might influence the results. Moreover, tissue inhomogenities are not regarded. However, with the help of MCX it should be possible to simulate random spatial variations (tissue inhomogenities).

The results might be even transferable to *in-vivo* spectroscopic data sets. For the oral cavity the sensitivity and specificity vary from around 65% to 100%^32^. For colon, the results strongly depend on the wavelength^17^ for ex-vivo tissue. An accuracy of 74% is reached for the visible light and an accuracy of 80% for NIR and accuracy of 91% for the combined data set. This leads to the conclusion that the limit of accuracy might also be due to the used spectral band. However, for the upper GI an accuracy of 85% could be reached by Goto *et al*.^20^ for an ex-vivo analysis from 400 to 800 nm. Thus, this conclusion cannot be done at the current state. Therefore, the lower accuracy of 68% in our older study^22^ might be caused by the *in-vivo* setting.

This is also a reason that current systems such as multi spectral light scattering devices^33^ are only tested on the biopsies. Even if the results in this study seem to be obvious, they still show that the multi spectral imaging process can be simulated by the means of MCS. Moreover with the usage of Bayesian statistics and the resulting a-posteriori probabilities as shown for diffuse reflection spectroscopy^28^, inter-patient variations might be simulations by means of MCS in the future for early studies of new optical approaches. Nevertheless, the next step would be animal studies to verify this study due to the fact that the animals can be dissected after the endoscopic procedure. With a successful verification of this method, MCS could be used to estimate the possible accuracy in future.

## Supplementary information

Supplementary Information.
