# Supplementary material for: Factors influencing the accuracy for tissue classification in multi spectral in-vivo endoscopy for the upper gastro-internal tract
Source: Sci Rep. 2020 Feb 26;10:3546. doi: 10.1038/s41598-020-60389-5 (PMC7044217; doi:10.1038/s41598-020-60389-5)
Supplement: Supplementary file 1 — Supplementary Information. [file 41598_2020_60389_MOESM1_ESM.pdf]

Factors influencing the accuracy for tissue classification in multi spectral in-vivo endoscopy for the upper gastro-internal tract - Supplementary Info

Martin Hohmann[1,2,*], Heinz Albrecht[3], Benjamin Lengenfelder[1,2], Florian Klämpfl[1,2], Michael Schmidt[1,2]

22nd January 2020

[1] Friedrich-Alexander-Universität Erlangen-Nürnberg (FAU), Institute of Photonic Technologies (LPT), Konrad-Zuse-Straße 3/5, 91052 Erlangen, Germany
[2] Erlangen Graduate School in Advanced Optical Technologies (SAOT), Paul-Gordan-Straße 6, 91052 Erlangen, Germany
[3] Kliniken des Landkreises Neumarkt i.d.OPf., Department of Internal Medicine II, Nürnberger Str. 12, 92318 Neumarkt, Germany
*correspond to: Martin.Hohmann@FAU.de

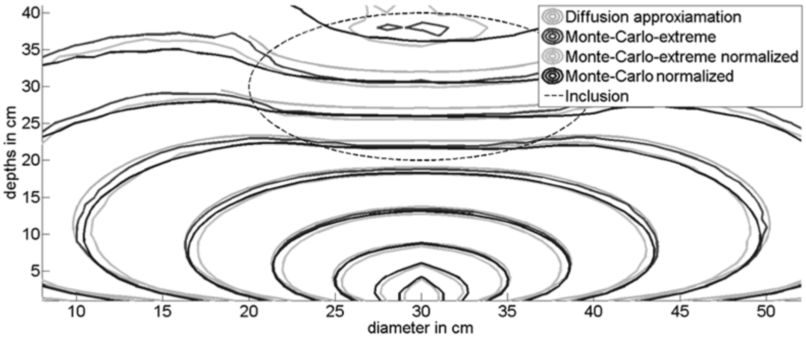

A: Validation results from the used MCS with diffusion approximation and MCX. The lines represent equipotential lines.

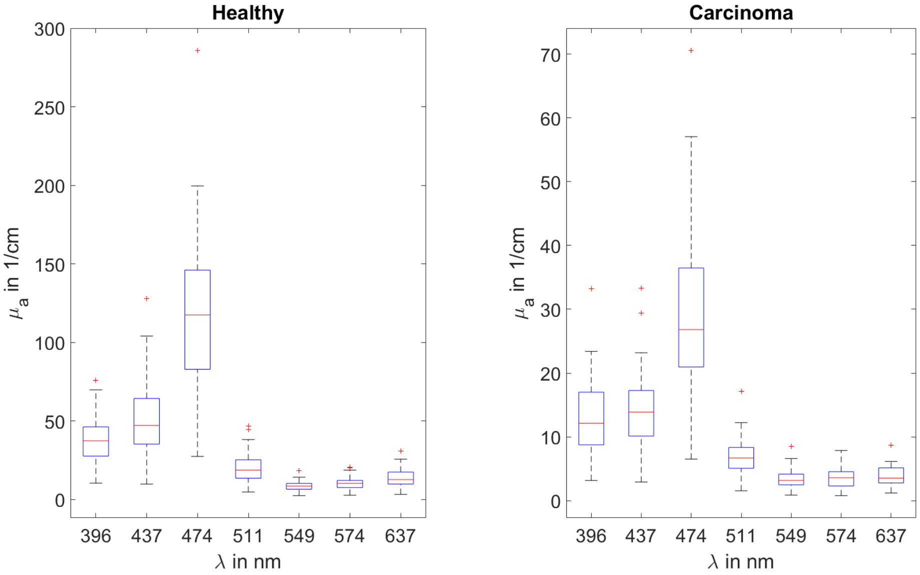

B: Boxplot of the random values used for the simulation of the absorption coefficient for the healthy and cancerous tissue.

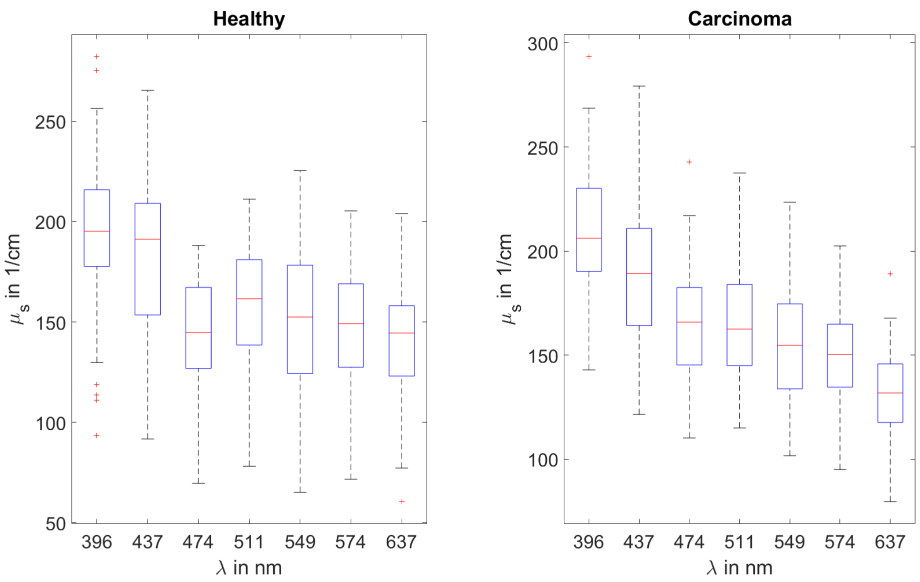

C: Boxplot of the random values used for the simulation of the scattering coefficient for the healthy and cancerous tissue.

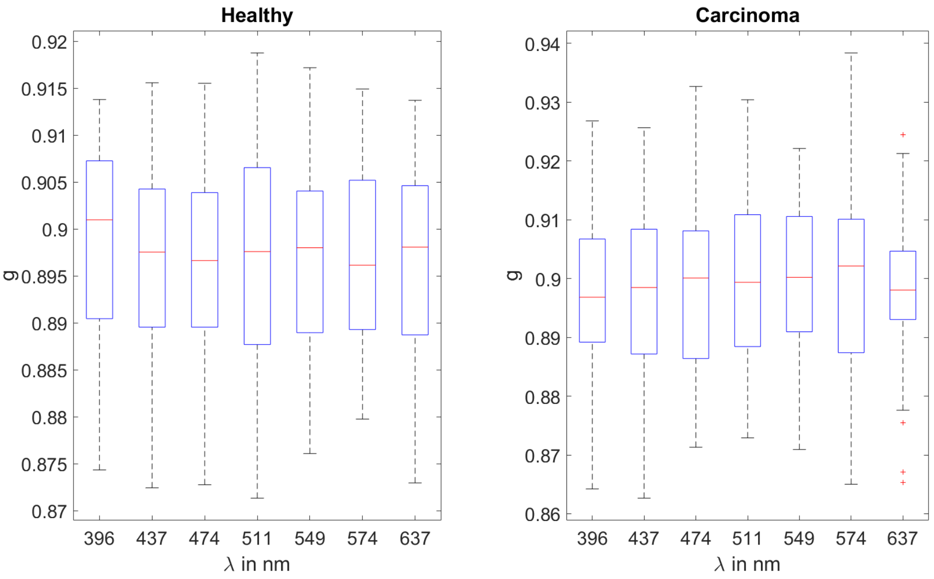

D: Boxplot of the random values used for the simulation of the g-factor for the healthy and cancerous tissue.
